# Lutein-Associated Crosstalk Between Hepatic Transcriptional Programs and Cecal Microbiota Is Linked to Antioxidant, Stress, and Immune Homeostasis in Laying Hens

**DOI:** 10.3390/antiox15060661

**Published:** 2026-05-24

**Authors:** Guanghui Li, Lei Liu, Hongchang Gu, Xia Chen, Zhixun Yan, Lingchao Zeng, Yutao Sun, Ying Bai, Huagui Liu, Qin Chu

**Affiliations:** 1 Institute of Animal Husbandry and Veterinary Medicine, Beijing Academy of Agriculture and Forestry Sciences, Beijing 100097, China; 2 College of Animal Science and Veterinary Medicine, Huazhong Agricultural University, Wuhan 430070, China; 3 Shenzhen Branch, Guangdong Laboratory of Lingnan Modern Agriculture, Key Laboratory of Livestock and Poultry Multi-Omics of MARA, Agricultural Genomics Institute at Shenzhen, Chinese Academy of Agricultural Sciences, Shenzhen 518124, China; 4 College of Life Science and Food Engineering, Hebei University of Engineering, Handan 056000, China; baiy019@126.com

**Keywords:** lutein, laying hens, liver transcriptome, cecal microbiota, host–microbiota interaction

## Abstract

Lutein is a dietary xanthophyll carotenoid with recognized antioxidant and immunomodulatory potential, yet the molecular basis underlying its nutritional effects in laying hens remains insufficiently understood. Here, liver transcriptomic profiling and 16S rRNA sequencing were combined to investigate the response of laying hens to dietary lutein supplementation. A total of 951 differentially expressed genes were identified in the liver, indicating marked transcriptional remodeling after lutein supplementation. Functional enrichment, gene set enrichment, and weighted gene co-expression network analyses consistently showed that these changes were mainly associated with metabolic regulation, redox/stress adaptation, and immune-related communication. In parallel, lutein supplementation changed cecal microbial community structure and shifted specific microbial biomarkers. Integrated correlation analyses further identified candidate host–microbiota association patterns, including a KLF2/*FOXO3*/*Faecalibacterium* axis and a KLF2/*IL8L2*/Prevotellaceae_Ga6A1_group axis. Overall, dietary lutein was associated with coordinated changes in the hepatic transcriptional profile and cecal microbial community structure, which converged on these two functional directions. These findings provide new insight into the nutritional effects of lutein in laying hens and identify candidate pathways and microbial nodes for future functional validation in poultry feeding systems.

## 1. Introduction

Lutein is a non-provitamin A xanthophyll carotenoid that must be obtained from the diet and is mainly found in green leafy vegetables, corn-based foods, and egg yolk [1,2,3]. It has long been studied because of its abundance in the human retina and because it is closely associated with macular pigment and visual health [3,4,5]. Beyond this classical role, lutein is now increasingly recognized as a dietary bioactive compound with broader functions in antioxidant defense, inflammatory regulation, and physiological homeostasis [6,7,8]. Recent reviews further suggest that carotenoids, including lutein, may also influence gut microbial ecology and intestinal function, extending interest in lutein from conventional nutrition to food bioactivity and host–microbiota regulation [9].

Laying hens provide a particularly relevant system for studying dietary lutein because they are both an important food-producing animal and an efficient biological vehicle for xanthophyll deposition into egg yolk. Previous studies have shown that dietary lutein can improve yolk pigmentation and support the production of lutein-enriched eggs with potential nutritional relevance for consumers [10,11]. Beyond egg pigmentation and lutein deposition, dietary lutein has also been reported to influence antioxidant status, immune responsiveness, reproductive performance, and hepatic lipid-related homeostasis in laying hens, although its effects on conventional laying performance and egg physical traits appear to be limited or variable among studies [12,13,14]. Mechanistically, this system is also informative because the liver is a central organ for lipid metabolism and yolk precursor synthesis in birds [14], whereas the cecum is one of the major sites of microbial fermentation in chickens [15,16]. Therefore, examining lutein responses in the liver and cecal microbiota may help explain how dietary bioactive compounds shape host metabolism and gut ecology in laying hens.

Emerging evidence further suggests that carotenoids may interact with intestinal microbiota. Studies in humans, animal models, and in vitro fermentation systems have shown that dietary carotenoids, including lutein, can affect microbial composition, microbial metabolic activity, and the production of organic acids [6]. In poultry, 16S rRNA sequencing has increasingly been used to evaluate how feed-derived bioactive compounds reshape cecal microbial communities. However, direct evidence linking lutein supplementation with cecal microbiota regulation in laying hens remains scarce. Therefore, integrating hepatic transcriptional responses with cecal microbiota changes may provide a more comprehensive view of the gut–liver implications of lutein supplementation.

Despite this progress, most poultry studies on lutein have primarily focused on yolk deposition, pigmentation, antioxidant or inflammatory biomarkers, reproductive traits, or single-layer molecular responses [14,17]. The current state of knowledge therefore remains fragmented: the hepatic transcriptional programs underlying lutein responses have not been fully characterized in laying hens, and the extent to which these host responses are associated with cecal microbial shifts remains unclear. In particular, it is unknown whether lutein-responsive hepatic genes and microbiota changes converge on shared functional directions, such as antioxidant/stress regulation and immune communication, within an integrated host–microbiota framework.

Therefore, this study investigated the coordinated effects of dietary lutein supplementation on the hepatic transcriptome and cecal microbiota of laying hens. Beijing-you laying hens were fed either a basal diet or a lutein-supplemented diet, followed by liver transcriptomic profiling and 16S rRNA sequencing of cecal contents. To move beyond single-gene or single-taxon comparisons, a series of in silico and network-based analyses were applied, including differential expression analysis, GO/KEGG enrichment analysis, GSEA, WGCNA, transcription factor-DEG correlation analysis, DEG–genus association analysis, and TF-DEG–genus Sankey integration. These approaches were used to identify pathway-level transcriptional programs, treatment-associated co-expression modules, and potential host–microbiota association patterns. This study aimed to provide a more comprehensive understanding of how dietary lutein modulates physiological responses in laying hens, with particular attention to Antioxidant Defense and Stress Response and Immune Regulation and Intercellular Communication.

## 2. Materials and Methods

### 2.1. Chemicals and Reagents

A lutein extract with a declared lutein concentration of 2.0% (*w*/*w*) was obtained from Chenguang Biotech Group Co., Ltd. (Handan, China). For the treatment diet, this preparation was added to the basal ration at 2.0% of the diet as the source of dietary lutein, resulting in a calculated final lutein concentration of approximately 400 mg/kg diet.

### 2.2. Experimental Animals, Dietary Treatment, and Sample Collection

A total of 120 clinically healthy Beijing You laying hens at 35 weeks of age were sourced from the Beijing You Chicken Conservation Farm (Beijing, China). The birds were randomly divided into two groups of equal size: a control group (Con) and a lutein-supplemented group (Lut). Hens assigned to the Con group received the basal diet, whereas those in the Lut group were given the same basal diet supplemented with 2.0% lutein extract containing 2.0% lutein, corresponding to a calculated final lutein concentration of approximately 400 mg/kg diet (Table 1). Because the hens were fed ad libitum, individual daily lutein intake was not directly measured; however, based on the calculated dietary concentration and an estimated feed intake of approximately 100–120 g/hen/day for adult laying hens, the estimated lutein intake was approximately 40–48 mg/hen/day. The nutrient composition of the experimental diets was calculated from the feed formulation, and the Con and Lut diets were formulated to be similar in metabolizable energy, crude protein, calcium, and nonphytate phosphorus. The feeding experiment was carried out over a period of 7 weeks. Throughout the trial, all birds were reared under the same husbandry conditions and had unrestricted access to feed and water. All animal procedures were reviewed and approved by the Institute of Animal Husbandry and Veterinary Medicine, Beijing Academy of Agriculture and Forestry Sciences (approval no. IHVM11-2402-71). Following the feeding period, eight hens from each group were randomly chosen for sampling and subjected to 12 h of fasting beforehand. After euthanasia, liver tissues and cecal contents were collected promptly, immediately frozen in liquid nitrogen, and then stored at −80 °C until subsequent analyses. The liver samples were used for transcriptome sequencing, while the cecal content samples were reserved for 16S rRNA gene sequencing.

### 2.3. RNA Isolation and Library Preparation

Transcriptome sequencing of liver samples was performed by Shanghai Majorbio Bio-pharm Technology Co., Ltd. (Shanghai, China). Total RNA was extracted from liver tissues with TRIzol Reagent (Invitrogen, Carlsbad, CA, USA) in accordance with the manufacturer’s protocol, followed by an additional purification step using an RNA Purification Kit supplied by Shanghai Majorbio Bio-pharm Technology Co., Ltd. (Shanghai, China). The quality of the isolated RNA was evaluated by agarose gel electrophoresis using Biowest Agarose (Biowest, Valencia, Spain). Only RNA samples meeting the requirements for integrity and library construction were retained for downstream sequencing. Libraries were generated with the Illumina Stranded mRNA Prep kit (Illumina, San Diego, CA, USA) according to the standard procedure and subsequently sequenced on an Illumina NovaSeq X Plus platform.

### 2.4. RNA-Seq Data Processing and Differential Expression Analysis

Raw reads were first subjected to quality control using fastp (v0.20.0) to remove adapter sequences and low-quality reads [18]. The resulting clean reads were then aligned to the chicken reference genome (GRCg6a) using HISAT2 (v2.1.0) [19], and gene expression levels were quantified with RSEM (v1.3.3) [20]. Across all libraries, the number of raw reads ranged from 43.65 to 58.43 million, whereas the number of clean reads ranged from 43.04 to 57.58 million after filtering. In addition, the proportion of bases with a Q30 quality score exceeded 93.67% in all samples, indicating that the sequencing data were of sufficient quality for subsequent transcriptomic analysis. Differential expression analysis between the Con and Lut groups was conducted using DESeq2 (v1.24.0) [21] in R (v4.5.3), based on the raw gene count matrix generated from RSEM quantification. DESeq2 normalization and model fitting were performed using the standard negative binomial framework, and statistical significance was evaluated using the Wald test. Genes with |log2FC| ≥ 1 and *p* < 0.05 were considered significantly differentially expressed genes (DEGs) and were used for subsequent functional enrichment and network analyses. The RNA-seq data generated in this study have been deposited in the Genome Sequence Archive (GSA) under accession number CRA041828 and are publicly available at https://ngdc.cncb.ac.cn/gsa/s/qW7fe069, accessed on 20 April 2026.

### 2.5. Functional Enrichment Analysis

To further interpret the biological functions and pathway associations of the identified DEGs, Gene Ontology (GO) and Kyoto Encyclopedia of Genes and Genomes (KEGG) enrichment analyses were performed. GO enrichment analysis was used to annotate DEGs at the levels of biological process, cellular component, and molecular function, whereas KEGG enrichment analysis was used to identify pathway-level changes; therefore, GO results were used to support and complement the biological interpretation of KEGG pathway enrichment. The DEG list was used as the input dataset, and all enrichment analyses were conducted using the clusterProfiler package (v4.6.2) [22] in R. For GO analysis, DEGs were annotated and classified into the categories of biological process (BP), cellular component (CC), and molecular function (MF), and terms with an adjusted *p* value < 0.05 were considered significantly enriched. KEGG pathway enrichment analysis was also performed using clusterProfiler, with *p* < 0.05 regarded as the threshold for significant enrichment. For data visualization, the enrichplot package (v1.18.4) in R was used to generate tree plots and gene-concept network plots of the significantly enriched GO terms and KEGG pathways. In addition, word clouds were generated to summarize the major enriched GO terms and KEGG pathways.

### 2.6. Gene Set Enrichment Analysis

Gene Set Enrichment Analysis (GSEA) was performed to identify coordinated functional changes at the gene set level. Prior to analysis, all genes were ranked in descending order according to their log2 fold change (log2FC) values between the Lut and Con groups. GSEA for GO terms and KEGG pathways was conducted using the relevant functions implemented in the clusterProfiler package in R, and enrichment plots were generated to visualize the significantly enriched gene sets. The normalized enrichment score (NES) was used to indicate the direction and relative magnitude of enrichment. Gene sets with |NES| > 1, nominal *p* value < 0.05, and P.adjust < 0.25 were considered significantly enriched. The P.adjust < 0.25 threshold was used as a conventional exploratory false discovery rate cutoff for GSEA to identify coordinated gene-set-level enrichment patterns. In this study, positive enrichment scores indicated gene sets enriched on the Lut side of the ranked gene list.

### 2.7. Weighted Gene Co-Expression Network Analysis

Weighted gene co-expression network analysis (WGCNA) was performed using the WGCNA package in R to identify coordinated transcriptional patterns associated with dietary lutein supplementation in the liver. Briefly, the normalized gene expression matrix was used as the input for network construction after removing genes with low expression levels. A soft-thresholding power was selected based on the scale-free topology criterion, and the resulting adjacency matrix was transformed into a topological overlap matrix (TOM). Genes were then hierarchically clustered according to TOM-based dissimilarity, and gene modules were identified using the dynamic tree cut method. Module eigengenes were calculated to represent the overall expression pattern of each module, and module–trait relationships were evaluated by correlating module eigengenes with the experimental groups. Modules significantly associated with lutein supplementation (*p* < 0.05) were considered key treatment-associated modules. Genes from these significant modules were subsequently subjected to GO and KEGG enrichment analyses using the clusterProfiler package in R to identify the major biological functions and pathways represented by each module.

### 2.8. Correlation Analysis and Key Transcription Factor (TF) Regulatory Network Construction

Spearman correlation analysis was performed in R to evaluate the associations between pathway-associated DEGs and transcription factors (TFs). In this study, TFs were first identified from the DEG set using the AnimalTFDB 4.0 database (https://guolab.wchscu.cn/AnimalTFDB4/#/, accessed on 20 April 2026), and only TFs that were themselves differentially expressed between the Con and Lut groups were retained for subsequent analysis. Correlation analyses were then conducted between these differentially expressed TFs and the DEGs collected from KEGG pathways representing two functional axes: Antioxidant Defense and Stress Response and Immune Regulation and Intercellular Communication. Only TF-DEG pairs with Spearman correlation *p* < 0.01 were retained for network construction. Based on these significant TF-DEG correlations, regulatory networks were further constructed using the online analysis platform of LC-Bio Technology Co., Ltd. (Hangzhou, China).

### 2.9. DNA Extraction, 16S rRNA Gene Amplification, and Sequencing

Cecal content samples were submitted to Shanghai Majorbio Bio-pharm Technology Co., Ltd. (Shanghai, China) for 16S rRNA gene sequencing. Microbial genomic DNA was extracted using the FastPure Fecal DNA Isolation Kit (magnetic bead-based; MJYH, Shanghai, China) according to the manufacturer’s instructions. The V3-V4 hypervariable region of the bacterial 16S rRNA gene was amplified using the primer pair 338F (5′-ACTCCTACGGGAGGCAGAG-3′) and 806R (5′-GGACTACHVGGGTWTCTAAT-3′). The resulting amplicons were purified and sequenced on an Illumina NextSeq 2000 platform (Illumina, San Diego, CA, USA) following the standard protocol provided by Shanghai Majorbio Bio-pharm Technology Co., Ltd.

### 2.10. Microbiota Data Processing and Differential Analysis

A total of 916,798 valid reads were obtained from the 16S rRNA sequencing dataset. Sequences were clustered into operational taxonomic units (OTUs) at 97% sequence similarity, resulting in a total of 2883 OTUs. Sequence processing and downstream analyses were carried out using the Majorbio Cloud Platform. Alpha diversity indices were calculated using mothur, whereas beta diversity was evaluated by principal coordinate analysis (PCoA) based on the Bray–Curtis distance. Community composition at different taxonomic levels was visualized using relative abundance bar plots. Differential taxa between the Con and Lut groups at the phylum and genus levels were assessed using the Wilcoxon rank-sum test, followed by Benjamini–Hochberg false discovery rate (FDR) correction for multiple comparisons. Taxa with FDR-adjusted *p* < 0.05 were considered significantly different. In addition, linear discriminant analysis effect size (LEfSe) was performed to identify taxa with differential abundance from the phylum to genus levels, using LDA > 3.5 and *p* < 0.05 as the screening criteria.

### 2.11. Correlation Analysis and Genus–Gene Association Network Construction

To explore potential gut–liver associations, integrated correlation analyses were performed using only paired samples for which both cecal microbiota and liver transcriptome data were available. Spearman correlation analysis was conducted in R to evaluate the associations between genus-level differential taxa and hepatic DEGs related to the two major functional directions identified in this study, namely Antioxidant Defense and Stress Response and Immune Regulation and Intercellular Communication. Correlation heatmaps were used to visualize the association patterns between differential genera and pathway-associated DEGs, and significant correlations were annotated with asterisks in the heatmaps. In addition, significant DEG–genus pairs filtered at *p* < 0.01 were further used for network construction and visualization using the online analysis platform of LC-Bio Technology Co., Ltd.

### 2.12. Statistics and Analysis

Unless otherwise specified, all statistical analyses were performed in R. For RNA-seq analysis, DEGs between the Con and Lut groups were identified using DESeq2, with |log2FC| ≥ 1 and *p* < 0.05 as the screening criteria. GO terms with adjusted *p* < 0.05 and KEGG pathways with *p* < 0.05 were considered significantly enriched. For GSEA, gene sets with |NES| > 1, nominal *p* < 0.05, and P.adjust < 0.25 were considered significantly enriched. For WGCNA, modules significantly associated with lutein supplementation were selected based on module–trait correlation *p* < 0.05. Microbial differential taxa were assessed using the Wilcoxon rank-sum test, and LEfSe biomarkers were identified using LDA > 3.5 and *p* < 0.05. Spearman correlation analysis was used for TF-DEG and DEG–genus association analyses, and only significant correlation pairs passing the thresholds specified in the corresponding sections were retained for network construction.

## 3. Results

### 3.1. Dietary Lutein Supplementation Reshaped the Hepatic Transcriptomic Profile of Laying Hens

**Figure 1:**
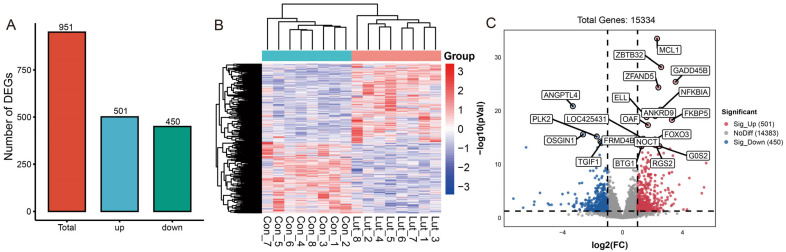
Differential expression analysis of hepatic genes in laying hens from the Con and Lut groups. (**A**) Number of DEGs identified between the Con and Lut groups, including total, up-regulated, and down-regulated genes. (**B**) Hierarchical clustering heatmap of hepatic DEGs in the Con and Lut groups. Each column represents one sample, and each row represents one gene. Colors indicate relative expression levels. (**C**) Volcano plot showing the distribution of hepatic genes between the Con and Lut groups. Red dots represent up-regulated genes, blue dots represent down-regulated genes, and gray dots represent genes without significant differential expression. Selected DEGs are labeled.

To obtain an overall view of the hepatic transcriptional response to dietary lutein supplementation, differential expression analysis was first performed between the Con and Lut groups. As shown in Figure 1A, a total of 951 DEGs were identified, including 501 up-regulated genes and 450 down-regulated genes in the Lut group compared with the Con group, indicating that lutein supplementation induced a marked transcriptional response in the liver. The hierarchical clustering heatmap further illustrated the overall expression patterns of these DEGs. As shown in Figure 1B, samples from the Con and Lut groups were clearly separated into two distinct clusters according to treatment, and the DEGs exhibited contrasting expression patterns between the two groups, suggesting an obvious difference in hepatic transcriptomic profiles after lutein supplementation.

### 3.2. GO Enrichment Analysis Highlighted Broad Functional Shifts in the Liver After Lutein Supplementation

**Figure 2:**
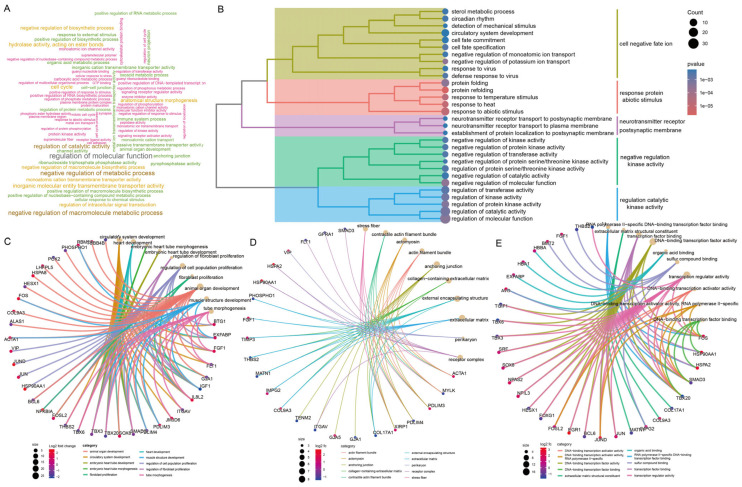
GO enrichment analysis of hepatic differentially expressed genes between the Con and Lut groups. (**A**) Word cloud summarizing the major enriched GO terms. (**B**) Tree plot showing the functional similarity relationships among enriched GO terms; dot size indicates gene count and color represents enrichment significance. (**C**–**E**) Gene-concept network plots of selected enriched GO terms in biological process (BP), cellular component (CC), and molecular function (MF), respectively. The size of each GO term node indicates the number of associated genes, and gene color represents log2 fold change.

To further characterize the biological functions associated with the hepatic differentially expressed genes identified above, Gene Ontology (GO) enrichment analysis was performed. As shown in Figure 2A, the enriched GO terms covered a broad range of biological themes, including regulation of catalytic activity, regulation of molecular function, negative regulation of metabolic process, monoatomic ion transmembrane transport, channel activity, response to stimulus, and organic/carboxylic acid metabolic processes. In addition, the tree plot grouped these enriched GO terms into several related functional clusters, mainly involving kinase/catalytic activity regulation, responses to protein folding and abiotic or viral stimuli, neurotransmitter receptor transport/localization-related processes, ion transport-associated regulation, and sterol metabolism/circadian rhythm-related terms (Figure 2B).

The gene-concept networks further showed that the enriched GO categories were mainly distributed in developmental and proliferative processes at the BP level, cytoskeletal and extracellular matrix-related structures at the CC level, and transcription factor-related activities and binding functions at the MF level (Figure 2C–E). Representative genes involved in these enriched categories included *FOS*, *JUN*, *JUND*, *BCL6*, *FLT1*, *FGF1*, *ITGAV*, *THBS2*, *SMAD3*, *ACTA1*, *MYLK*, *COL9A3*, *HSP90AA1*, and *TBX20*, indicating that dietary lutein supplementation was associated with broad changes in hepatic biological regulation, structural organization, and transcription-related functions in laying hens.

### 3.3. KEGG Enrichment Analysis Revealed Coordinated Pathway Remodeling in the Liver After Lutein Supplementation

**Figure 3:**
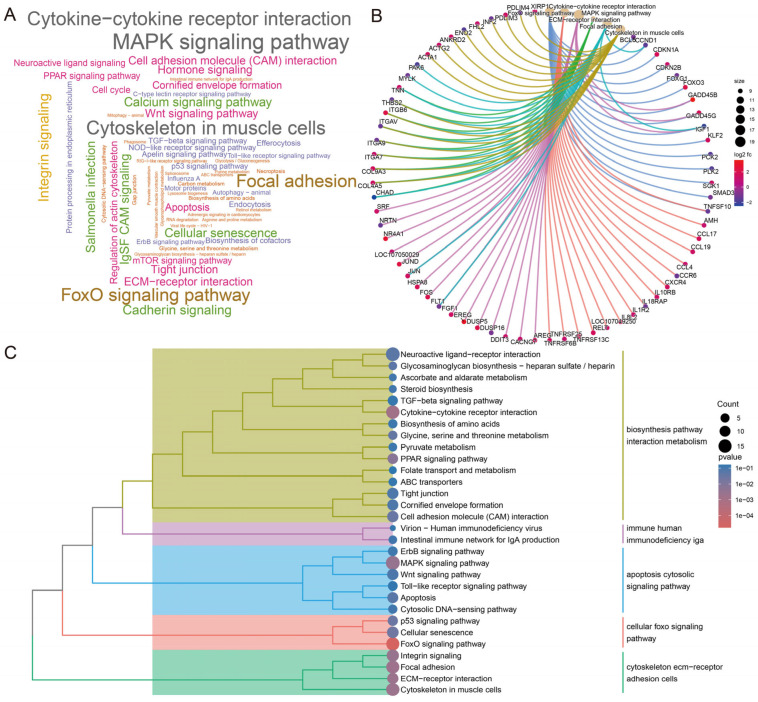
KEGG enrichment analysis of hepatic differentially expressed genes between the Con and Lut groups. (**A**) Word cloud summarizing the major enriched KEGG pathways. (**B**) Gene-concept network showing the relationships between the top 6 enriched KEGG pathways and their associated differentially expressed genes. The size of each pathway node indicates the number of associated genes, and gene color represents log2 fold change. (**C**) Tree plot showing the functional similarity and clustering of the top 30 enriched KEGG pathways; dot size indicates gene count and color represents enrichment significance.

Following the GO analysis, KEGG enrichment analysis was performed to further identify the signaling and metabolic pathways associated with the hepatic DEGs. As shown in Figure 3A, the enriched pathways mainly involved signal transduction, immune- and stress-related responses, cell adhesion and cytoskeleton organization, and metabolic regulation, including FoxO signaling pathway, cytokine–cytokine receptor interaction, MAPK signaling pathway, focal adhesion, ECM–receptor interaction, cytoskeleton in muscle cells, PPAR signaling pathway, pyruvate metabolism, and biosynthesis of amino acids.

The gene-concept network further showed that these enriched pathways were interconnected through a series of representative DEGs (Figure 3B). For example, genes such as *FOXO3*, *GADD45B*, *GADD45G*, *CDKN1A*, *CDKN2B*, and *IGF1* were associated with the FoxO signaling pathway and related cellular regulatory processes, whereas *CCL17*, *CCL19*, *CCL4*, *CCR6*, *CXCR4*, *IL10RB*, *IL18RAP*, *IL1R2*, and *IL8L2* were mainly enriched in cytokine–cytokine receptor interaction. In addition, *FOS*, *JUN*, *JUND*, *DUSP5*, *DUSP16*, *FGF1*, and *FLT1* were involved in the MAPK signaling pathway, while *ITGA7*, *ITGA9*, *ITGAV*, *ITGB6*, *COL4A5*, *COL9A3*, *THBS2*, *MYLK*, *PDLIM3*, and *XIRP1* were linked to ECM–receptor interaction, focal adhesion, and cytoskeleton in muscle cells.

As shown in Figure 3C, the enriched KEGG pathways could be further grouped into several major functional clusters, mainly including metabolism- and biosynthesis-related pathways, immune- and infection-related pathways, apoptosis- and intracellular stress signaling-related pathways, cellular regulatory pathways centered on FoxO signaling, and pathways related to the cytoskeleton, extracellular matrix, and cell adhesion. Notably, several of these pathways, including FoxO signaling pathway, MAPK signaling pathway, p53 signaling pathway, apoptosis, and some immune-related pathways, are closely associated with oxidative stress responses and inflammatory regulation. Together, these findings suggest that lutein supplementation induced coordinated remodeling of hepatic pathways related to metabolism, signaling transduction, immune-associated processes, and structural organization.

### 3.4. GSEA Revealed Directional Differences in Hepatic Functional Programs Between the Con and Lut Groups

**Figure 4:**
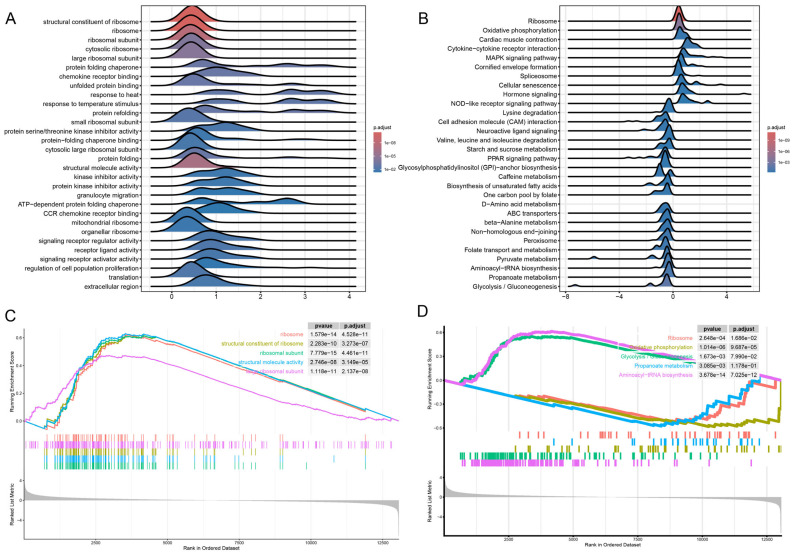
GSEA of hepatic transcriptomic changes between the Con and Lut groups. (**A**) Ridge plot showing the top 30 enriched GO gene sets identified by GSEA. (**B**) Ridge plot showing the top 30 enriched KEGG pathways identified by GSEA. (**C**) Enrichment plots of the top 5 GO terms. Positive enrichment indicates gene sets enriched on the Lut side of the ranked gene list. (**D**) Enrichment plots of the top 5 KEGG pathways. Positive enrichment indicates pathways enriched in the Lut group, whereas negative enrichment indicates pathways enriched in the Con group.

To further determine the directional enrichment patterns of hepatic functional changes induced by lutein supplementation, GSEA was performed for both GO terms and KEGG pathways. The top 30 GO gene sets were mainly related to ribosome- and translation-associated functions, protein folding and refolding, chaperone activity, receptor binding, and stress-related responses, including heat and temperature responses (Figure 4A). The top 30 KEGG pathways were mainly involved in ribosome, oxidative phosphorylation, glycolysis/gluconeogenesis, pyruvate metabolism, aminoacyl-tRNA biosynthesis, propanoate metabolism, PPAR signaling, peroxisome, and several immune- and signaling-related pathways, such as cytokine–cytokine receptor interaction and MAPK signaling (Figure 4B).

As shown in Figure 4C, the top five GO gene sets, including ribosome, structural constituent of ribosome, ribosomal subunit, structural molecule activity, and large ribosomal subunit, all showed positive enrichment scores, indicating that these ribosome- and translation-related functions were enriched on the Lut side of the ranked gene list. This result suggests that dietary lutein supplementation was associated with increased enrichment of ribosome-associated structural and translational gene sets in the liver.

In contrast, the top five KEGG pathways exhibited a bidirectional enrichment pattern (Figure 4D). Glycolysis/Gluconeogenesis and Aminoacyl-tRNA biosynthesis showed positive enrichment and were therefore enriched on the Lut side of the ranked gene list, whereas Ribosome, Oxidative phosphorylation, and Propanoate metabolism showed negative enrichment and were enriched on the Con side. Thus, the GSEA results do not indicate a simple global activation of protein synthesis or energy metabolism by lutein. Instead, they suggest a pathway-dependent hepatic transcriptional shift, with lutein-associated enrichment of selected substrate metabolism- and aminoacyl-tRNA-related programs and relative depletion of several ribosome-, oxidative phosphorylation-, and propanoate metabolism-related gene sets. These directional changes provide transcriptomic evidence for hepatic adaptive remodeling, but they should be interpreted together with enrichment and network analyses rather than as direct biochemical proof of altered protein synthesis or oxidative metabolism.

### 3.5. WGCNA Identified Co-Expression Modules Associated with Lutein Supplementation

**Figure 5:**
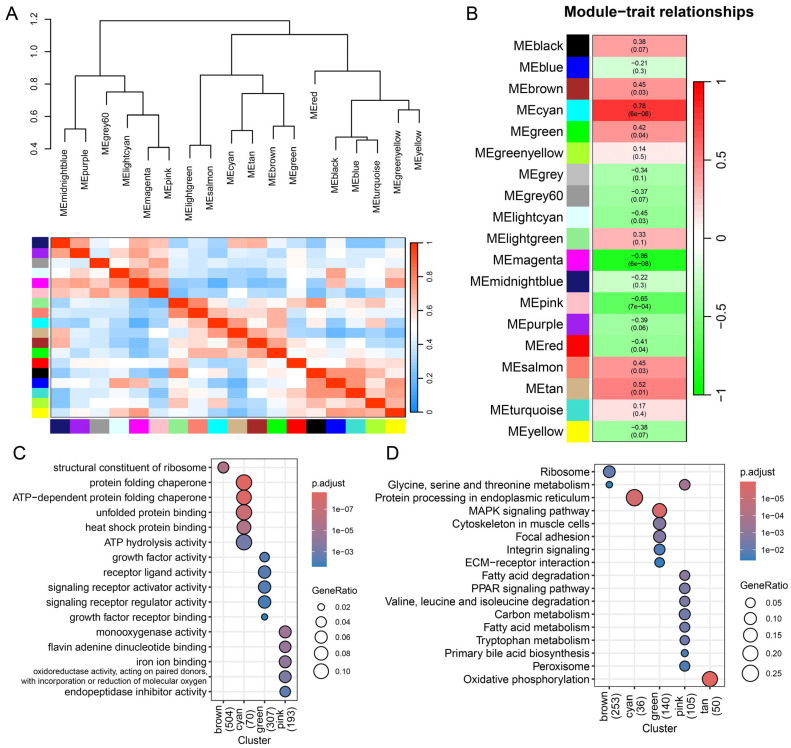
WGCNA of hepatic transcriptomic data and functional enrichment of core modules. (**A**) Clustering dendrogram and eigengene adjacency heatmap of co-expression modules identified by WGCNA. (**B**) Module–trait relationship heatmap showing the correlations between module eigengenes and the treatment trait. Correlation coefficients are shown in each cell, with corresponding *p* values in parentheses. (**C**) Dotplot of the top five GO terms enriched in the selected core modules. (**D**) Dotplot of the top ten KEGG pathways enriched in the selected core modules.

Because coordinated transcriptional responses often involve groups of co-regulated genes rather than isolated DEGs, WGCNA was further conducted to identify treatment-associated gene modules. As shown in Figure 5A, multiple co-expression modules were identified and clustered according to eigengene similarity. The module–trait relationship heatmap further showed that several modules were significantly associated with the treatment trait (Figure 5B), including MEbrown (*r* = 0.45, *p* = 0.03), MEcyan (*r* = 0.78, *p* = 6 × 10^−6^), MEgreen (*r* = 0.42, *p* = 0.04), MEpink (*r* = −0.65, *p* = 7 × 10^−4^), and MEtan (*r* = 0.52, *p* = 0.01). Among them, MEcyan showed the strongest positive correlation, whereas MEpink showed a marked negative correlation with the treatment trait. In addition, MEmagenta also exhibited a strong negative association (*r* = −0.86, *p* = 6 × 10^−8^) (Figure 5B).

To further characterize the biological functions of the core modules, enrichment analyses were performed. The top five GO terms of the selected core modules were mainly associated with structural constituent of ribosome, protein folding chaperone, ATP-dependent protein folding chaperone, unfolded protein binding, heat shock protein binding, growth factor activity, receptor ligand activity, monooxygenase activity, flavin adenine dinucleotide binding, iron ion binding, and oxidoreductase activity (Figure 5C). These results indicate that the core modules were mainly related to ribosome-associated structure, protein folding and chaperone functions, receptor/growth factor-related activity, and oxidation-related molecular functions.

KEGG enrichment analysis of the selected core modules further showed that the top ten enriched pathways were mainly involved in oxidative phosphorylation, peroxisome, primary bile acid biosynthesis, tryptophan metabolism, fatty acid metabolism, carbon metabolism, valine, leucine and isoleucine degradation, PPAR signaling pathway, fatty acid degradation, ECM–receptor interaction, integrin signaling, focal adhesion, cytoskeleton in muscle cells, MAPK signaling pathway, protein processing in endoplasmic reticulum, glycine, serine and threonine metabolism, and ribosome (Figure 5D). Overall, these findings suggest that treatment-associated co-expression modules were not linked to a single isolated pathway, but rather to coordinated hepatic programs involving protein folding/chaperone activity, oxidoreductase-related functions, peroxisome and PPAR-related lipid metabolism, energy-substrate metabolism, and extracellular matrix/cell-adhesion signaling. Therefore, the WGCNA results were used to prioritize lutein-associated transcriptional modules for biological interpretation, rather than to infer that lutein directly induced de novo protein synthesis.

### 3.6. Transcription Factor Correlation Analysis Revealed Multilevel Associations Under Antioxidant Defense and Stress Response and Immune Regulation and Intercellular Communication

**Figure 6:**
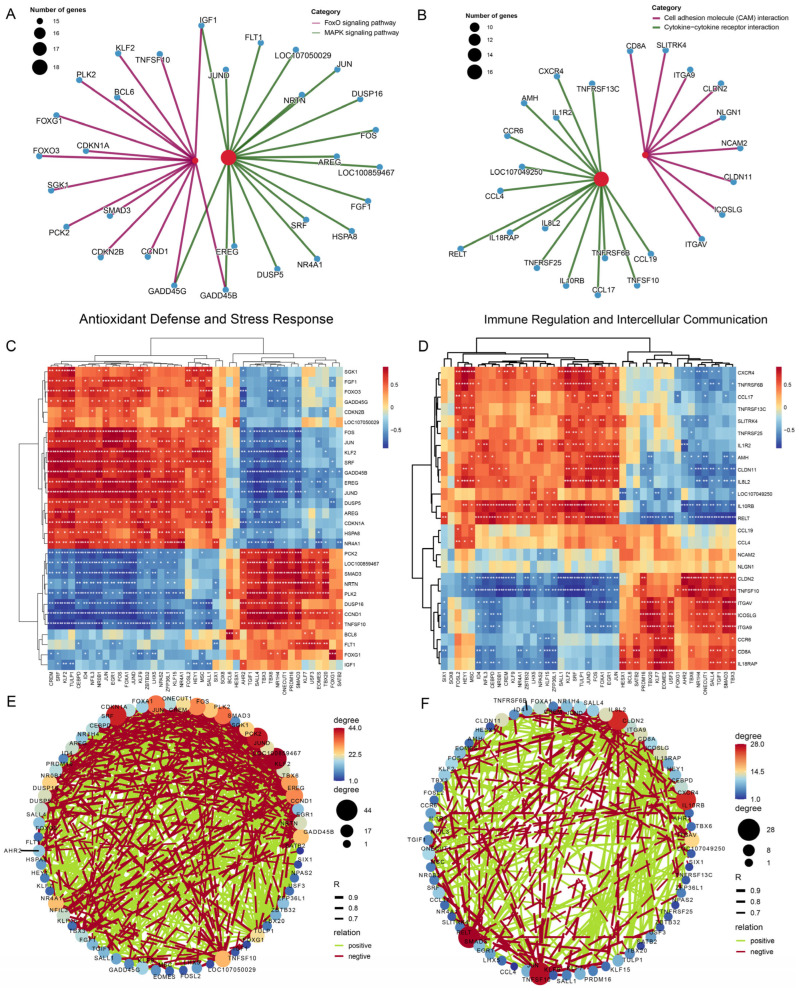
TF-DEG association analysis of hepatic gene sets related to Antioxidant Defense and Stress Response and Immune Regulation and Intercellular Communication. (**A**) DEGs extracted from FoxO signaling pathway and MAPK signaling pathway, representing the Antioxidant Defense and Stress Response direction. (**B**) DEGs extracted from cytokine–cytokine receptor interaction and cell adhesion molecule interaction, representing the Immune Regulation and Intercellular Communication direction. (**C**,**D**) Correlation heatmaps showing associations between differentially expressed transcription factors and pathway-associated DEGs under the two functional directions; * *p* < 0.05, ** *p* < 0.01, *** *p* < 0.001. (**E**,**F**) Simplified TF-DEG association networks constructed from significant correlations filtered at *p* < 0.01. Node size reflects degree, and edge color indicates positive or negative correlation. For clarity, representative high-degree TFs and key pathway-associated DEGs are highlighted in the network plots. These networks indicate candidate TF-DEG association patterns rather than experimentally validated regulatory relationships.

Based on the pathway and module-level results above, TF-centered association analyses were performed to identify candidate transcriptional regulators linked to the two major functional directions. For the Antioxidant Defense and Stress Response direction, DEGs from FoxO signaling pathway and MAPK signaling pathway were extracted because these pathways were closely related to redox/stress-responsive signaling (Figure 6A). For the Immune Regulation and Intercellular Communication direction, DEGs from cytokine–cytokine receptor interaction and cell adhesion molecule interaction were collected because these pathways represented immune signaling and intercellular communication processes (Figure 6B). These curated DEG sets were then used for TF-DEG correlation analysis.

Correlation clustering analysis was then performed between these pathway-associated DEGs and TFs that were themselves differentially expressed between the Con and Lut groups. As shown in Figure 6C,D, both the Antioxidant Defense and Stress Response and Immune Regulation and Intercellular Communication gene sets exhibited extensive significant correlations with the selected TFs, and the heatmaps revealed clear clustered patterns of both positive and negative associations. In the Antioxidant Defense and Stress Response direction, genes such as *SGK1, FOXO3*, *GADD45B*, *GADD45G*, *CDKN1A*, *CDKN2B*, *PCK2*, *SMAD3*, *PLK2*, *DUSP5*, *DUSP16*, and *TNFSF10* showed broad correlations with multiple DE TFs (Figure 6C). In the Immune Regulation and Intercellular Communication direction, genes including *CXCR4*, *TNFRSF6B*, *TNFRSF13C*, *TNFRSF25*, *IL1R2*, *IL10RB*, *IL18RAP*, *CCL17*, *CCL19*, *CCL4*, *CCR6*, *CD8A*, *ITGA9*, *ITGAV*, *CLDN2*, and *CLDN11* also displayed widespread correlations with multiple DE TFs (Figure 6D).

To further summarize these relationships, simplified TF-DEG association networks were constructed using significant correlation pairs filtered at *p* < 0.01. As shown in Figure 6E,F, the revised networks highlight representative high-degree TFs and key pathway-associated DEGs under the two functional directions. These networks should be interpreted as correlation-based association maps rather than direct evidence of transcriptional regulation. Taken together, these results suggest that lutein-responsive TFs were associated with selected stress-, signaling-, immune-, and communication-related DEGs, thereby providing candidate TF-DEG links for subsequent host–microbiota integration analyses.

### 3.7. Lutein Supplementation Changed Cecal Microbial Community Structure in Laying Hens

**Figure 7:**
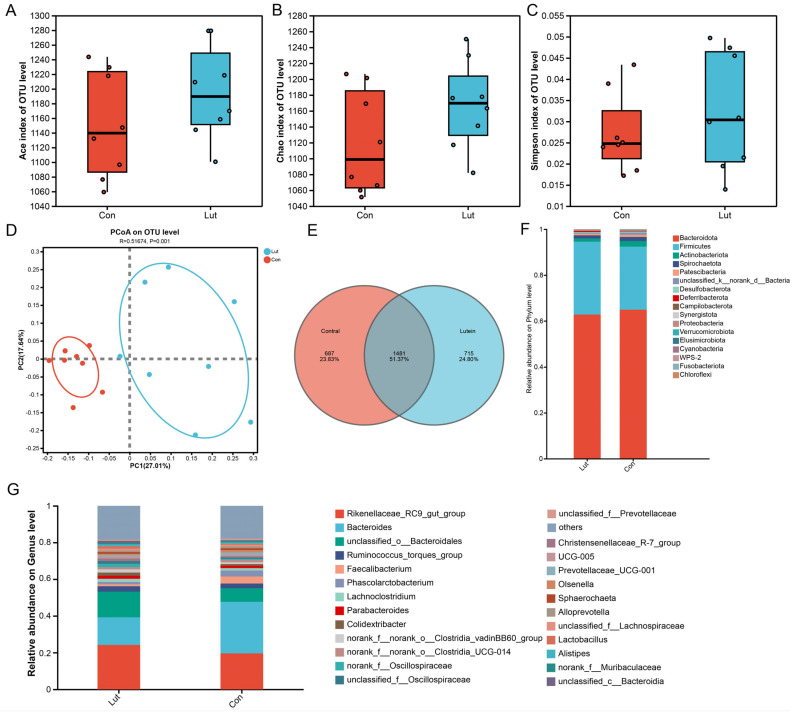
Effects of dietary lutein supplementation on the cecal microbial community of laying hens. (**A**–**C**) Alpha diversity indices at the OTU level, including Ace, Chao, and Simpson indices. (**D**) Principal coordinates analysis (PCoA) of cecal microbial communities at the OTU level. (**E**) Venn diagram showing shared and unique OTUs between the Con and Lut groups. (**F**) Relative abundance of cecal microbiota at the phylum level. (**G**) Relative abundance of cecal microbiota at the genus level.

In addition to the hepatic transcriptomic changes, the effects of lutein supplementation on cecal microbial communities were further investigated. As shown in Figure 7A–C, the Ace, Chao, and Simpson indices showed overlapping distributions between the Con and Lut groups, although the Lut group generally exhibited higher median values than the Con group.

At the community structure level, principal coordinates analysis (PCoA) based on OTUs showed separation between the Con and Lut groups (Figure 7D), with *r* = 0.51674 and *p* = 0.001, indicating that lutein supplementation was associated with a detectable shift in cecal microbial structure. Consistently, the Venn diagram showed that the two groups shared 1481 OTUs (51.37%), whereas 687 OTUs (23.83%) were unique to the Con group and 715 OTUs (24.80%) were unique to the Lut group (Figure 7E).

At the phylum level, the cecal microbiota of both groups was dominated by Bacteroidota and Firmicutes, followed by lower-abundance phyla such as Actinobacteriota, Spirochaetota, Patescibacteria, Desulfobacterota, Proteobacteria, Verrucomicrobiota, and Fusobacteriota (Figure 7F). At the genus level, the major taxa included Rikenellaceae_RC9_gut_group, *Bacteroides*, unclassified_o__Bacteroidales, Ruminococcus_torques_group, *Faecalibacterium*, *Phascolarctobacterium*, *Lachnoclostridium*, *Parabacteroides*, and *Colidextribacter* (Figure 7G). Overall, these results indicate that dietary lutein supplementation altered the cecal microbial community structure of laying hens.

### 3.8. Lutein Supplementation Changed the Abundance of Specific Cecal Microbial Taxa

**Figure 8:**
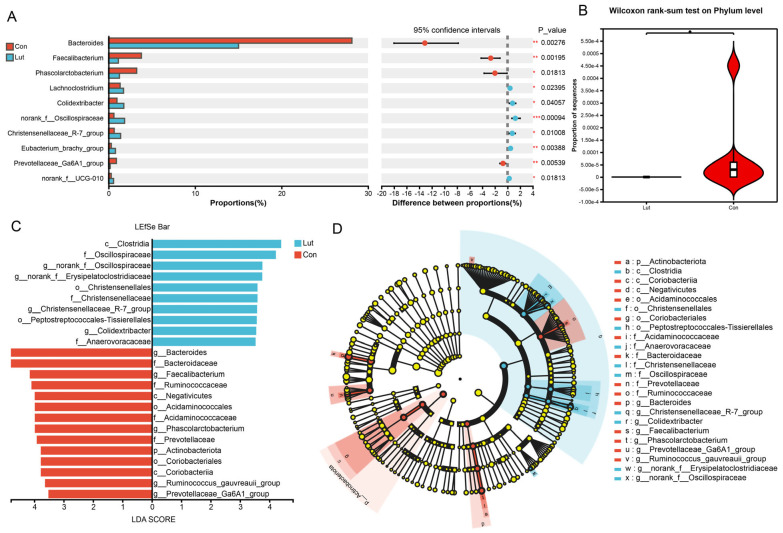
Differential taxonomic analysis and LEfSe identification of cecal microbial biomarkers between the Con and Lut groups. (**A**) Wilcoxon rank-sum analysis of the significantly differential genera between the Con and Lut groups. The left panel shows relative abundance, and the right panel shows the difference between proportions with 95% confidence intervals and corresponding *p* values. (**B**) Wilcoxon rank-sum analysis at the phylum level. Fusobacteriota was the only significantly differential phylum between the two groups (*p* < 0.05). (**C**) LEfSe bar plot showing taxa with significant differential enrichment between the Con and Lut groups based on LDA scores. (**D**) LEfSe cladogram showing the phylogenetic distribution of differentially enriched taxa between the two groups, * *p* < 0.05, ** *p* < 0.01, *** *p* < 0.001.

To further define the microbial taxa responsible for the group separation observed above, differential taxonomic analyses were performed at the genus and phylum levels, followed by LEfSe analysis. At the genus level, Wilcoxon rank-sum analysis identified 10 significantly different genera between the Con and Lut groups (Figure 8A). Among them, *Bacteroides, Faecalibacterium, Phascolarctobacterium*, and *Prevotellaceae_Ga6A1_group* were more abundant in the Con group, whereas *Lachnoclostridium, Colidextribacter,* norank_f__Oscillospiraceae, Christensenellaceae_R-7_group, Eubacterium_brachy_group, and norank_f__UCG-010 were more abundant in the Lut group.

At the phylum level, only one taxon showed a significant difference between the two groups. As shown in Figure 8B, *Fusobacteriota* was the only significantly differential phylum, and its relative abundance was higher in the Con group than in the Lut group. LEfSe analysis further identified microbial biomarkers that discriminated the two groups (Figure 8C,D). Taxa enriched in the Lut group were mainly assigned to the Clostridia-Oscillospiraceae-Christensenellaceae-related lineages, including *c__Clostridia, f__Oscillospiraceae*, *g__norank_f__Oscillospiraceae*, *o__Christensenellales*, *f__Christensenellaceae*, *g__Christensenellaceae_R-7_group*, *o__Peptostreptococcales-Tissierellales*, *g__Colidextribacter*, and *f__Anaerovoracaceae*. In contrast, taxa enriched in the Con group included p__Actinobacteriota, c__Coriobacteriia, o__Coriobacteriales, c__Negativicutes, o__Acidaminococcales, f__Acidaminococcaceae, f__Bacteroidaceae, g__Bacteroides, g__Faecalibacterium, g__Phascolarctobacterium, f__Prevotellaceae, and g__Prevotellaceae_Ga6A1_group. Overall, these results indicate that lutein supplementation altered the differential taxonomic composition of the cecal microbiota and was associated with a shift in microbial biomarkers.

### 3.9. Integrated Correlation Analysis of DEGs and Genus-Level Microbiota Associated with Antioxidant Defense and Stress Response and Immune Regulation and Intercellular Communication

**Figure 9:**
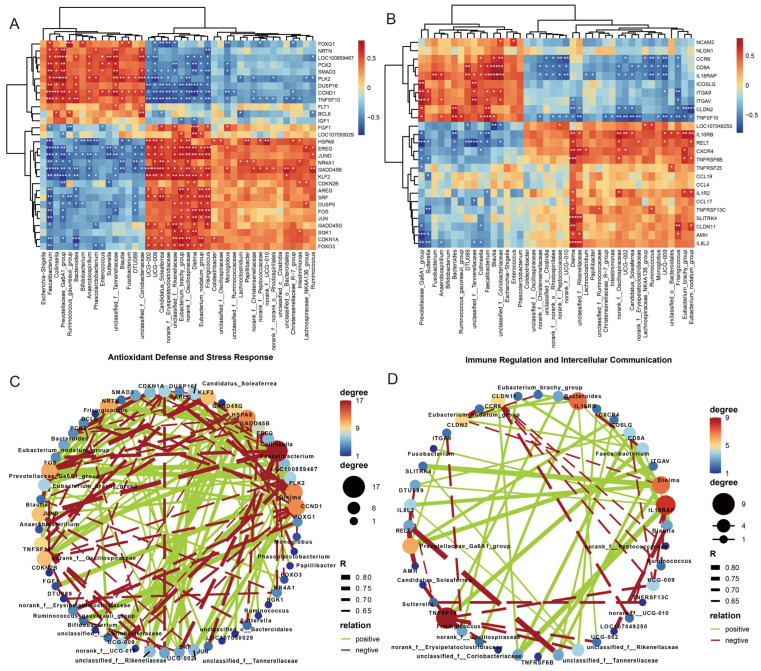
Correlation analysis between differential genera and DEGs associated with Antioxidant Defense and Stress Response and Immune Regulation and Intercellular Communication. (**A**) Correlation clustering heatmap between genus-level differential taxa and DEGs associated with Antioxidant Defense and Stress Response, * *p* < 0.05, ** *p* < 0.01, *** *p* < 0.001. (**B**) Correlation clustering heatmap between genus-level differential taxa and DEGs associated with Immune Regulation and Intercellular Communication, * *p* < 0.05, ** *p* < 0.01, *** *p* < 0.001. (**C**) Correlation network of DEG–-genus pairs associated with Antioxidant Defense and Stress Response, filtered at *p* < 0.01. (**D**) Correlation network of DEG–genus pairs associated with Immune Regulation and Intercellular Communication, filtered at *p* < 0.01. Edge color indicates positive or negative correlation, and node size reflects degree.

To further investigate host–microbiota associations under the two major functional directions, correlation analyses were performed between genus-level differential taxa and DEGs associated with Antioxidant Defense and Stress Response and Immune Regulation and Intercellular Communication, respectively. As shown in Figure 9A,B, both heatmaps revealed extensive positive and negative correlations between differential genera and pathway-associated DEGs, indicating close associations between cecal microbial shifts and hepatic transcriptional responses under these two functional directions.

In the Antioxidant Defense and Stress Response direction, DEGs such as *FOXO3*, *CDKN1A*, *SGK1*, *GADD45B*, *GADD45G*, *FOS*, *JUN*, *JUND*, *NR4A1*, *DUSP5, DUSP16*, *PLK2*, *PCK2*, and *SMAD3* showed broad correlations with multiple differential genera, including *Bacteroides, Faecalibacterium, Prevotellaceae_Ga6A1_group*, *Phascolarctobacterium*, *Eubacterium_brachy_group*, *norank_f__Oscillospiraceae*, *Lachnoclostridium*, and *Christensenellaceae_R-7_group* (Figure 9A). In the Immune Regulation and Intercellular Communication direction, genes such as *IL18RAP*, *ITGAV*, *CD8A*, *ICOSLG*, *CXCR4*, *TNFRSF6B*, *TNFRSF13C*, *CCL19*, *CCL4*, *IL1R2*, *CCL17*, and *IL8L2* also exhibited widespread correlations with multiple genera, including *Bacteroides*, *Faecalibacterium*, *Prevotellaceae_Ga6A1_group*, *Fusobacterium*, *Dielma*, *DTU089*, *Eubacterium_brachy_group*, and *norank_f__Oscillospiraceae* (Figure 9B).

To further summarize these associations, correlation networks were constructed using the DEG–genus pairs filtered at *p* < 0.01. As shown in Figure 9C,D, both functional directions formed dense interaction networks containing both positive and negative correlations. The network associated with Antioxidant Defense and Stress Response appeared more complex, with a higher maximum node degree than that of Immune Regulation and Intercellular Communication, suggesting a broader pattern of DEG–genus associations in this direction. Overall, these results indicate that lutein supplementation was associated with coordinated host–microbiota relationships involving DEGs and differential genera linked to Antioxidant Defense and Stress Response and Immune Regulation and Intercellular Communication.

### 3.10. Integrated TF-DEG–Genus Sankey Analysis Revealed Multilevel Association Patterns Under Two Major Functional Directions

**Figure 10:**
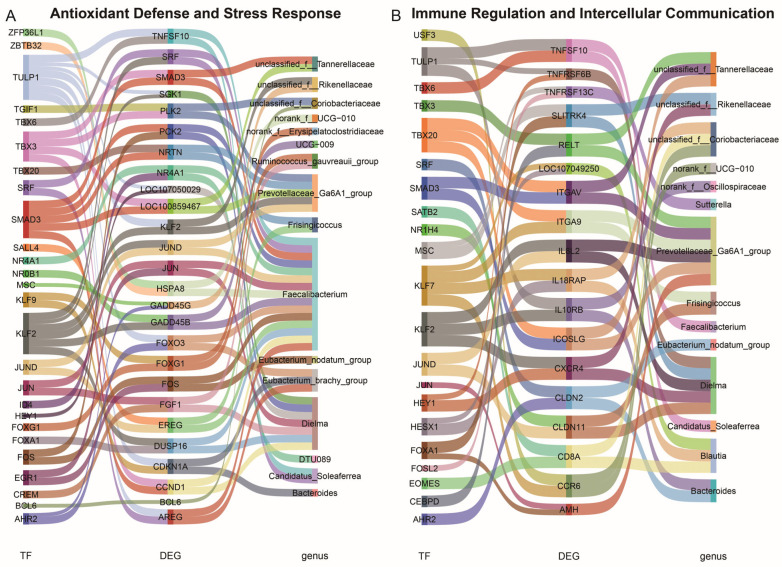
TF-DEG–genus Sankey diagrams under Antioxidant Defense and Stress Response and Immune Regulation and Intercellular Communication. (**A**) Sankey diagram showing the integrated associations among differentially expressed transcription factors, DEGs, and genus-level microbiota related to Antioxidant Defense and Stress Response. (**B**) Sankey diagram showing the integrated associations among differentially expressed transcription factors, DEGs, and genus-level microbiota related to Immune Regulation and Intercellular Communication.

To integrate the transcription factor-gene and gene–microbiota associations identified above, TF-DEG–genus Sankey diagrams were constructed for the two major functional directions using associations filtered at *p* < 0.01. As shown in Figure 10A, the Sankey diagram for Antioxidant Defense and Stress Response integrated lutein-responsive transcription factors, pathway-associated DEGs, and genus-level microbiota into a continuous three-layer association framework. Notably, this network highlighted a KLF2/*FOXO3/Faecalibacterium* axis, suggesting a candidate multilevel association linking stress-related hepatic transcriptional variation with a microbial node.

Similarly, the Sankey diagram for Immune Regulation and Intercellular Communication also revealed a clear multilevel association pattern connecting differentially expressed TFs, immune-related DEGs, and genus-level microbiota (Figure 10B). In this direction, the network highlighted a KLF2/IL8L2/Prevotellaceae_Ga6A1_group axis, suggesting a candidate association between immune-related transcriptional variation and microbial shifts.

Overall, these Sankey diagrams highlighted distinct host–microbiota association features under the two major functional directions, with a *KLF2/FOXO3/Faecalibacterium* axis in Antioxidant Defense and Stress Response and a *KLF2/IL8L2/Prevotellaceae_Ga6A1_group* axis in Immune Regulation and Intercellular Communication. These results further support that dietary lutein supplementation was associated with coordinated shifts in TFs, DEGs, and genus-level microbiota, and also help prioritize key multilevel association nodes for subsequent biological interpretation.

## 4. Discussion and Conclusions

Dietary lutein supplementation induced coordinated changes in both the hepatic transcriptome and cecal microbiota of laying hens. Hepatic transcriptomic analyses revealed broad functional remodeling involving metabolism, signaling pathways, and stress- and immune-associated biological processes, whereas 16S rRNA sequencing showed a clear shift in cecal microbial community structure accompanied by changes in specific microbial biomarkers. Previous studies in chickens have similarly shown that dietary lutein can improve antioxidant status, alter liver gene expression and metabolites, and attenuate inflammatory responses, while broader evidence from other systems also supports its role in redox and immune regulation [17,23,24,25]. More importantly, the integrated analyses in this study indicated that these host and microbial changes converged on two major functional directions, namely Antioxidant Defense and Stress Response and Immune Regulation and Intercellular Communication. The TF-DEG, DEG–genus, and TF-DEG–genus association networks further supported the view that the biological effects of lutein were characterized by coordinated host–microbiota interactions rather than isolated transcriptomic or microbial changes. Importantly, these findings should not be interpreted as evidence that lutein nonspecifically activates all major cellular pathways. Rather, in the present study, lutein appeared to act as a dietary bioactive factor associated with coordinated adjustment of hepatic redox/stress-responsive programs, lipid and substrate metabolism, cytokine- and adhesion-related communication, and cecal microbial community structure. Moreover, the present data do not demonstrate that lutein acts selectively on the liver. The liver was selected because it is a central organ for lipid metabolism, yolk precursor synthesis, and carotenoid-related metabolic responses in laying hens, whereas cecal microbiota were analyzed to explore potential gut–liver associations. Therefore, the current results should be regarded as a systems-level, hypothesis-generating framework that prioritizes specific pathways and host–microbiota axes for future functional validation, rather than direct evidence that lutein selectively induces protein synthesis or acts only in the liver.

One of the most prominent transcriptomic features after lutein supplementation was the remodeling of hepatic programs related to Antioxidant Defense and Stress Response. This interpretation is supported by the enrichment of pathways such as FoxO signaling, MAPK signaling, apoptosis, oxidative phosphorylation, and peroxisome, together with WGCNA- and GSEA-derived signals involving oxidoreductase activity, protein folding/chaperone functions, and stress-responsive biological processes. These findings suggest that the primary hepatic signature associated with lutein supplementation was not the activation of a single antioxidant pathway, but a coordinated adaptation of redox-sensitive signaling, protein quality control, and energy/lipid-related metabolism in the liver. A previous integrative transcriptomic and metabolomic study in chickens similarly linked dietary lutein to antioxidant improvement, altered hepatic gene expression, and lipid metabolism-related processes [17]. Broader mechanistic evidence from other systems also suggests that lutein can modulate redox-sensitive signaling and endogenous antioxidant defenses rather than functioning only as a direct radical scavenger [25]. Accordingly, the concurrent enrichment of PPAR signaling, pyruvate metabolism, glycolysis/gluconeogenesis, and fatty acid metabolism in our data suggests that hepatic antioxidant defense may be accompanied by parallel adjustments in substrate utilization and energy metabolism.

In addition to Antioxidant Defense and Stress Response, the hepatic response to lutein was also strongly associated with Immune Regulation and Intercellular Communication. This was reflected by the enrichment of cytokine–cytokine receptor interaction and cell adhesion molecule (CAM) interaction, together with the coordinated changes in immune- and adhesion-related genes identified in the integrated analyses. These findings suggest that the hepatic response to lutein extended beyond oxidative adaptation and involved coordinated modulation of cytokine signaling, receptor-mediated communication, and cell–cell interaction processes that are important for immune homeostasis. Poultry studies support this interpretation, as lutein supplementation has been shown to alleviate inflammatory responses, improve mucosal barrier integrity, and suppress TLR4/MyD88/NF-κB-related signaling under challenge conditions [23]. Evidence from non-poultry systems further strengthens this view, indicating that lutein can attenuate pro-inflammatory cytokines and modulate immune-associated oxidative balance across tissues and species [26]. Taken together, these observations suggest that lutein shapes hepatic immune-associated transcriptional programs within a broader regulatory framework rather than affecting isolated inflammatory signals alone.

The transcriptional remodeling induced by lutein was accompanied by a clear restructuring of the cecal microbiota. Notably, the dominant microbial signal in this dataset was not a simple change in richness-related indices, but a distinct shift in community structure, as indicated by the OTU-based PCoA separation together with differential taxa and microbial biomarkers. This pattern suggests that lutein influenced microbial community organization and compositional balance rather than merely increasing or decreasing overall diversity. A similar phenomenon has been reported in laying hens fed xanthophyll-rich orange corn, supporting the plausibility that carotenoid-rich dietary interventions can reshape cecal microbial structure in poultry [27]. More broadly, current evidence indicates that carotenoids can interact with gut microbial ecosystems, although the magnitude and direction of these effects likely depend on carotenoid type, host background, and dietary context [28]. Therefore, the microbial shifts observed here are more appropriately interpreted as an ecologically relevant component of the overall lutein response than as isolated taxonomic changes. A key question is why lutein supplementation was associated with a shift in cecal bacterial composition. Several possible mechanisms may explain this observation. First, lutein is a lipophilic dietary carotenoid, and its intestinal absorption is unlikely to be complete; therefore, part of the supplemented lutein or lutein-containing dietary matrix may reach the lower intestine and cecum, where it could influence the local luminal environment and microbial ecological niches. Second, lutein may indirectly affect the cecal microbiota by modulating host redox status, immune tone, lipid-related metabolism, bile-acid-associated conditions, or substrate availability, all of which can influence bacterial community structure. Third, in the present study, the altered genera were not interpreted as isolated taxonomic changes, because DEG–genus and TF-DEG–genus analyses linked microbial variation with hepatic genes involved in oxidative/stress responses, immune-related communication, and metabolic regulation. Therefore, the observed bacterial shift may reflect a broader diet-associated host–microbiota response to lutein supplementation. However, these mechanisms remain hypothetical, and the present 16S rRNA sequencing data cannot determine whether lutein directly acted on bacteria or indirectly reshaped the microbial ecosystem through host-mediated physiological changes.

An important feature of this study is that lutein-responsive changes could be organized into integrated host–microbiota association patterns. The TF-DEG, DEG–genus, and TF-DEG–genus analyses consistently suggested that the hepatic response to lutein was accompanied by coordinated associations with genus-level microbial variation, rather than representing independent changes on either side. In this sense, the present work extends previous carotenoid-related studies by showing that the effects of lutein in laying hens can be interpreted not only through hepatic pathways or cecal taxa alone, but also through multilevel association networks linking transcription factors, DEGs, and microbiota. This perspective is consistent with the emerging concept of holo-omics, which treats host transcriptional programs and microbiome configurations as interconnected components of the same biological system [29]. Notably, the two major functional directions displayed different microbial association features. *Faecalibacterium* emerged as a prominent node within the Antioxidant Defense and Stress Response network, suggesting a close association with host transcriptional changes related to redox balance and stress adaptation. In contrast, *Prevotellaceae_Ga6A1_group* was more strongly connected within the Immune Regulation and Intercellular Communication network, indicating that it may represent a key microbial association node linked to immune-related transcriptional variation. This interpretation is biologically plausible because *Faecalibacterium* is widely recognized as an anti-inflammatory commensal in other systems, whereas *Prevotellaceae_Ga6A1_group* has been repeatedly reported as a cecal taxon associated with fermentation-related microbial ecology in poultry [30,31]. These findings help prioritize specific microbial nodes for subsequent functional investigation, although the current evidence remains correlation-based rather than causal.

A further implication of these integrated networks is that they point to more specific multilevel association axes that may help explain how lutein-responsive host and microbial changes are coordinated. In both functional directions, *KLF2* emerged as a shared transcriptional node, which is notable because *KLF2* is widely regarded as a homeostatic transcription factor involved in restraining inflammatory activation and regulating chemokine receptor- and adhesion-related programs [32]. Within the Antioxidant Defense and Stress Response direction, the *KLF2/FOXO3/Faecalibacterium* axis is particularly interesting, as *FOXO3* is a well-established stress-responsive transcription factor that contributes to oxidative stress resistance, mitochondrial homeostasis, apoptosis, and autophagy-related adaptation [33,34]. This suggests that the association between *KLF2* and *FOXO3* may represent an upstream transcriptional framework linked to the antioxidant and stress-related microbial pattern identified in our study. By contrast, in the Immune Regulation and Intercellular Communication direction, the *KLF2/IL8L2/Prevotellaceae_Ga6A1_group* axis may reflect a distinct immune-associated linkage, given that *IL8L2* is a chicken chemokine closely related to early innate inflammatory signaling and leukocyte recruitment [35]. Taken together, these two axes provide a more refined interpretation of the host–microbiota association patterns revealed by the present study, although their biological relevance will still require targeted functional validation in future work.

Several limitations should also be acknowledged. First, the TF-DEG, DEG–genus, and TF-DEG–genus networks were constructed based on correlation analyses; therefore, they should be interpreted as association-based models rather than causal regulatory pathways. Second, qRT-PCR validation of representative DEGs was not performed in the present study, and protein-level or biochemical validation was also not included. Therefore, the transcriptomic findings should be regarded as sequencing-based evidence that requires further experimental confirmation. Third, productive performance and egg quality data were not included in the current analysis, which limits our ability to directly connect the observed omics changes with laying performance or egg-related phenotypes. Future studies should include qRT-PCR validation, protein or enzyme activity assays, oxidative and immune biomarkers, productive performance traits, egg quality traits, and dose-response designs to confirm the biological and physiological relevance of the candidate pathways and host–microbiota axes identified here.

Overall, dietary lutein supplementation was associated with coordinated alterations in hepatic transcriptional programs and cecal microbial structure in laying hens, and these changes converged on two major functional directions, namely Antioxidant Defense and Stress Response and Immune Regulation and Intercellular Communication. Rather than demonstrating that lutein directly or selectively activates protein synthesis in the liver, the present multi-omics results support a systems-level model in which dietary lutein is linked to hepatic redox/stress adaptation, immune-related communication, and specific host–microbiota association axes. These findings deepen current understanding of lutein biology in poultry and provide candidate pathways and microbial nodes for future mechanistic validation and nutritional application in laying hen feeding systems. However, because this study used a single supplementation level and association-based multi-omics analyses, the dose dependency, causality, and functional consequences of these host–microbiota changes require further investigation.

## Figures and Tables

**Table 1:** Composition and nutrient levels of the experimental diets.

Term	Con	Lut
Ingredients		
Corn	62.00	62.00
Soybean meal	24.00	24.00
Limestone	9.20	9.20
Choline chloride (50%)	0.12	0.12
Sodium chloride	0.3	0.3
Dicalcium phosphate	1.15	1.15
Maifan stone	2.86	0.86
L-lysine sulfate	0.05	0.05
DL-methionine	0.15	0.15
Phytase (10,000 IU)	0.02	0.02
Trace mineral premix for laying hens ^1^	0.10	0.10
Vitamin premix for laying hens ^2^	0.05	0.05
Lutein ^3^	-	2.00
Total	100.00	100.00
Nutrient levels ^4^		
Metabolizable energy (MJ/kg)	11.50	11.50
Crude protein (%)	16.00	16.00
Calcium (%)	3.00	3.00
Nonphytate phosphorus (%)	0.36	0.36

Notes: ^1^ Trace mineral premix for laying hens. ^2^ Vitamin premix for laying hens. ^3^ The calculated final lutein concentration in the Lut diet was approximately 400 mg/kg diet. Independent chemical/proximate analysis of the experimental diets was not performed in the present study. ^4^ Nutrient levels were calculated values.

## Data Availability

The original data presented in the study are openly available in the Genome Sequence Archive (GSA) at https://ngdc.cncb.ac.cn/gsa/s/qW7fe069, accession number CRA041828, accessed on 20 April 2026.
